# Why humans build fires shaped the same way

**DOI:** 10.1038/srep11270

**Published:** 2015-06-08

**Authors:** Adrian Bejan

**Affiliations:** 1 Duke University, Department of Mechanical Engineering and Materials Science, Durham, North Carolina 27708-0300, USA

## Abstract

Here we see why humans unwittingly build fires that look the same: edifices of fuel, as tall as they are wide. The pile of fuel is permeable, air invades it by natural convection and drives the combustion. I show that the hottest pile of burning fuel occurs when the height of the pile is roughly the same as its base diameter. Future studies may address the shape effect of wind, material type, and packing. Key is why humans of all eras have been relying on this design of fire “unwittingly”. The reason is that the heat flow from fire facilitates the movement and spreading of human mass on the globe.

Our bonfires are shaped as cones and pyramids, as tall as they are wide at the base. They look the same in all sizes, from the firewood in the chimney, to the tree logs and wooden benches in the center of the university campus after the big game. They look the same as the pile of charcoal we make to grill meat.

Why is this human preference for burning piles that look the same? Why do we build the same “edifice” of burning fuel every time? Why do we do it instinctively, without having to learn it in school or steal the idea from the neighbor?

Fire was good for movement (life), and because it was good it was adopted^1^,^2^,^3^,^4^,^5^,^6^,^7^. The adoption of fire in human civilization was a design change (a transition) of the same nature as the emergence of organs for vision in animal design, the emergence of turbulence in laminar flow, the emergence of terrestrial animal locomotion from swimming, and the emergence of flying later on. This step occurred in an unmistakable direction, from no fire to fire, not the other way around. Why?

The answer is the same for all the transitions: to facilitate movement and mixing on earth. For us, fire means ultimately more movement for humanity on the landscape, in accord with the constructal law^8^,^9^. Fire accounts for many empowering features that enable the human mass to move more easily and for greater access. Controlled fire is a human contrivance for instant and portable shelter, which is good for the continuity of movement. With fire the early humans did not depend on caves for warmth, dryness and safety.

The shapes of piles of coal, stones, sand and other materials have attracted considerable attention in physics. The stability of piles of granular materials^10^ and the ‘fibrous’ distribution of forces through the material^11^ depends on pile shape. The wind and the packing density have a significant effect on the self-heating of coal piles^12^ and on erosion^13^. The shape and size of a pile of stones governs the total work spent on constructing the pile^14^. Although the fundamentals of fluid mechanics concerning flow through coarse deformable granular piles (e.g., fluidized beds) and permeable interfaces with ambient have received attention^15^,^16^,^17^, the effect of the shape of the burning pile on its temperature and heating performance was not questioned until now.

## Results

**Figure 1:**
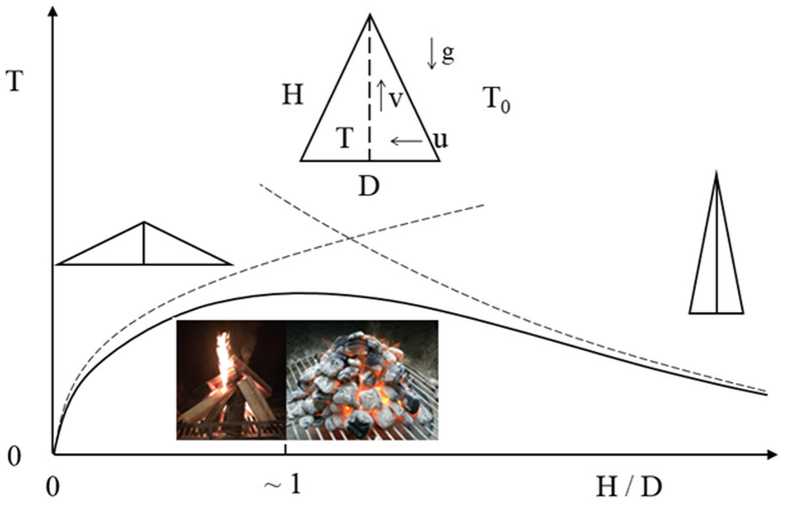
The fire temperature (T) as a function of the shape of the profile of the pile of fuel. The clashing asymptotes define this behavior, and the method of intersecting the asymptotes^18^ pinpoints the architecture, the design. Photographs taken by Adrian Bejan.

Here is why our fires should be built to look the same. Consider the model of a burning pile of fuel as a volume V of height H, base dimension D, and absolute temperature T. The volume is illustrated as a cone or pyramid in Fig. 1, but it can have any shape (such as a parallelepiped), provided that the height of the body is H, and that the base area has a single length scale D (for example, round or square). The scale analysis reported below does not capture the effect of volume shape, cone versus pyramid, except for the aspect ratio of the volume profile, H/D.

The body of fuel is a porous medium through which fluid flows because of the buoyancy effect due to low density (hot) fluid inside the body, and high density (cold) fluid outside the body. For a hot volume of height H, the pressure difference scale that drives fluid through the cone structure of temperature T is^18^


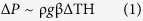


where ρ is the air density, averaged between the air outside and inside, β is the coefficient of volumetric thermal expansion, ΔT is the temperature difference T − T_0_, where T_0_ is the ambient temperature, and g is the gravitational acceleration.

The flow of air and combustion gases through the body is modeled as Darcy flow through a saturated porous medium with known permeability K, which scales with the pore diameter squared^18^. The heat transfer from the surface of the body to the ambient is modeled as black body radiation. We consider this flow in the two extremes, a tall pile (H 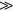 D), and a shallow pile (H 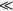 D). The treatment is based on scale analysis, which means that we neglect dimensionless factors of order 1.

### Tall limit

The volume averaged velocity of the fluid that permeates through the body is oriented in the vertical direction,


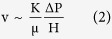


where μ is the fluid viscosity, which is treated as constant. Combining Eqs. (1) and (2) we find that v ~ Kgβ ΔT/ν, where ν is μ/ρ. The scale of the mass flow rate through the tall porous body is


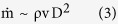


where D^2^ is the scale of the body cross sectional area. The heat generated by combustion inside the body is proportional to the flow rate of oxidant 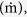 therefore we represent it as 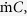 where C accounts for the reaction of combustion and the heating value of the fuel^19^. The heat generation rate 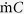 is transferred to the ambient (T_0_) via radiation,


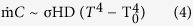


with the observation that σ is the Stefan-Boltzmann constant, and HD is the scale of the external surface of the tall body. Combining Eqs. (3) and (4), and noting that when T > T_0_ we can approximate ΔT ~ T and T^4^ – 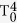 ~ T^4^, and obtain the scale of the body temperature


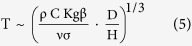


This result shows that the body temperature decreases as the body becomes taller. In practice, needed is a high temperature, therefore a shorter body is of interest. This is why we turn our attention to the opposite extreme:

### Shallow limit

In a shallow porous body the volume averaged fluid velocity is oriented horizontally,


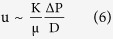


The mass flow rate is 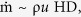 where HD is the scale of the cross sectional area pierced by u. The radiation heat transfer rate is 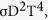 where D^2^ is the scale of the external surface of the body. In place of Eq. (4) we write 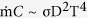 and obtain the scale of the shallow body temperature,


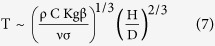


This shows that the shallow design is also inferior, because the body becomes colder as it becomes more shallow.

### Intersection of asymptotes

Together, the two extremes covered by the preceding analysis are represented by the two asymptotes sketched in Fig. 1. The actual curve that relates T to the aspect ratio H/D is a bell-shaped curve that fits under the intersection of the two asymptotes. Most useful is the design that offers the hottest fire. This design is easy to identify by finding the H/D location of the intersection of the two asymptotes, Eqs. (5) and (7) which is


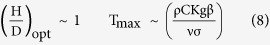


The best edifice of fuel must be such that its height is comparable with its diameter at the base. This geometric conclusion is independent of the thermophysical properties that were necessary in constructing the model of the flow phenomenon (g, ρ, μ, ν, K, σ, C, β).

Alternatively, the analysis outlined between Eqs. (2)–(8), can be repeated while regarding the volume of fuel as fixed (V ~ D^2^H), and searching for the height H, or the base length D, for which T is at its peak. After the intersection of the tall and shallow asymptotes, the conclusion is that H_opt_ ~ V^1/3^, or D_opt_ ~ V^1/3^, is the same as in Eq. (8).

This conclusion does not change if we replace β with T^–1^, which is recommended by the ideal gas model for the fluid^19^. The scale of the peak temperature achieved when H ~ D is found from either Eq. (5) or Eq. (7), with β = T^–1^,


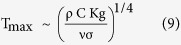


This confirms common knowledge: the fire is hotter when it “breathes” better, which happens when the permeability K increases.

## Discussion

A fundamental result such as Eq. (9) also reveals aspects of behavior that are not accessible to the human senses. The temperature of the hottest fire increases in proportion with g^1/4^, therefore the same fire will be hotter in a higher-g environment, in a centrifuge rotating at high speed, or on a planet just like Earth but larger.

Important to keep in perspective is that the model described in the preceding analysis is perhaps the simplest imaginable. The model is an invitation to more focused studies aimed at additional effects that may affect the pile shape sensibly: the wind speed and direction, the type of fuel (coal, wood), and the packing (fine, coarse). For example, if the packing is coarse enough, the Darcy flow model will give way to more refined models^20^ that can be implemented numerically. If the wind is strong enough, the radiation heat transfer from the pile will be replaced by a conjugate radiation & convection heat transfer model.

## Additional Information

**How to cite this article**: Bejan, A. Why humans build fires shaped the same way. *Sci. Rep.* **5**, 11270; doi: 10.1038/srep11270 (2015).
